# Vasopressor Use for Severe Hypotension—A Multicentre Prospective Observational Study

**DOI:** 10.1371/journal.pone.0167840

**Published:** 2017-01-20

**Authors:** Francois Lamontagne, Deborah J. Cook, Maureen O. Meade, Andrew Seely, Andrew G. Day, Emmanuel Charbonney, Karim Serri, Yoanna Skrobik, Paul Hebert, Charles St-Arnaud, Hector Quiroz-Martinez, Michaël Mayette, Daren K. Heyland

**Affiliations:** 1 Department of Medicine, Université de Sherbrooke, Sherbrooke, Quebec, Canada; 2 Centre de Recherche du CHU de Sherbrooke, Sherbrooke, Quebec, Canada; 3 Department of Medicine, McMaster University, Hamilton, Ontario, Canada; 4 Department of Clinical Epidemiology & Biostatistics, McMaster University, Hamilton, Ontario, Canada; 5 Thoracic Surgery and Critical Care Medicine, University of Ottawa, Ottawa, Ontario, Canada; 6 Ottawa Hospital Research Institute, Ottawa, Ontario, Canada; 7 Clinical Evaluation Research Unit, Kingston General Hospital, Kingston, Ontario, Canada; 8 Centre de Recherche de l’Hôpital du Sacré-Coeur de Montréal, University of Montreal, Montreal, Canada; 9 Centre hospitalier affilié universitaire régional de Trois-Rivières; 10 McGill University, Montreal, Quebec, Canada; 11 Department of Medicine, Centre Hospitalier de l'Université de Montréal, Montreal, Quebec, Canada; University of PECS Medical School, HUNGARY

## Abstract

**Background:**

The optimal approach to titrate vasopressor therapy is unclear. Recent sepsis guidelines recommend a mean arterial pressure (MAP) target of 65 mmHg and higher for chronic hypertensive patients. As data emerge from clinical trials comparing blood pressure targets for vasopressor therapy, an accurate description of usual care is required to interpret study results. Our aim was to measure MAP values during vasopressor therapy in Canadian intensive care units (ICUs) and to compare these with stated practices and guidelines.

**Method:**

In a multicenter prospective cohort study of critically ill adults with severe hypotension, we recorded MAP and vasopressor doses hourly. We investigated variability across patients and centres using multivariable regression models and Analysis of variance (ANOVA), respectively.

**Results:**

We included data from 56 patients treated in 6 centers. The mean (standard deviation [SD]) age and Acute Physiology and Chronic Health Evaluation (APACHE) II score were 64 (14) and 25 (8). Half (28 of 56) of the patients were at least 65 years old, and half had chronic hypertension. The patient-averaged MAP while receiving vasopressors was 75 mm Hg (6) and the median (1^st^ quartile, 3^rd^ quartile) duration of vasopressor therapy was 43 hours (23, 84). MAP achieved was not associated with history of underlying hypertension (p = 0.46) but did vary by center (p<0.001).

**Conclusions:**

In this multicenter, prospective observational study, the patient-level average MAP while receiving vasopressors for severe hypotension was 75 mmHg, approximately 10 mmHg above current recommendations and stated practices. Moreover, our results do not support the notion that clinicians tailor vasopressor therapy to individual patient characteristics such as underlying chronic hypertension but MAP achieved while receiving vasopressors varied by site.

## Introduction

In shock, hypotension may lead to hypoperfusion, organ failure and death.[1, 2] Vasopressors are administered under the assumption that vasoconstriction will improve organ perfusion when hypotension is caused by abnormal vasodilation.[3–5] However, vasoconstriction may be excessive and hinder blood flow to vital organs in an unpredictable fashion, especially if concomitant abnormal cardiac function and hypovolemia compound vasodilation. Vasopressors also carry the risk of various adverse effects. [6, 7] The balance between risks and benefits of alternative vasopressor dosing strategies may vary between patients.

Practice guidelines suggest that chronic hypertension may warrant higher mean arterial pressure (MAP) targets.[8] Physicians appear to agree with these recommendations and underlying assumptions. In a survey of stated practices among more than 200 Canadian intensive care specialists, a MAP of 65 mmHg was the most common initial target and 88% of respondents stated that they aimed for higher MAP in patients with chronic hypertension.[9] A retrospective review of documented targets for vasopressor titration for 369 patients in Canada and Australia[10] found a median initial MAP target of 65 mmHg, concordant with stated practices. However, in this study, chronic hypertension was not associated with higher target MAP.

The aim of this study was to measure actual MAP values among hypotensive patients receiving vasopressor therapy. The secondary objective was to measure associations between baseline characteristics (age, chronic hypertension, congestive heart failure) and MAP values.

## Methods

We conducted a multicenter prospective observational study. The Research Ethics Board of participating hospitals approved the protocol (i.e. Centre intégré universitaire de santé et de services sociaux de l'Estrie—formally the Centre hospitalier universitaire de Sherbrooke—St. Joseph's Healthcare Hamilton, Ottawa Health Science Network, Hôpital Maisonneuve-Rosement, Centre hospitalier affilié universitaire régional de Trois-Rivières, and the Centre intégré universitaire de santé et de services sociaux du Nord-de-l'Île-de-Montréal—formally Hôpital du Sacré-Coeur de Montréal). Dedicated research teams screened patients for eligibility in each intensive care unit (ICU). Upon confirming eligibility, they obtained written consent from the patients or their legal representatives. When patients were incapacitated and their representatives were unavailable, we used a deferred consent model.

Using web-based enrollment, we included adults (≥16 years of age) receiving vasopressor therapy for severe hypotension (as defined by treating team) that persisted after adequate fluid resuscitation, defined as at least 30 mL/kg, or a perception of the treating physician that additional fluid could be harmful. Based on the judgment of treating physicians, we limited eligibility to patients expected to remain on vasopressors for at least 6 hours. We excluded patients who received vasopressors to treat intracranial hypertension or angioedema, as well as patients who were unstable following cardiopulmonary bypass, acute left ventricular failure or hemorrhage.

Research personnel at each centre recorded MAP values and vasopressor doses hourly. When more than one measure was available for a given 1-hour interval, we used the average of the highest and lowest values. Blood pressure measurements were typically transduced from an arterial catheter and recorded in the electronic (or paper) medical record, which we used as source documents. We transformed dopamine, epinephrine, phenylephrine and vasopressin doses to norepinephrine-equivalents, as previously described.[11] We recorded patient characteristics at baseline (age, sex, weight, height, primary admission diagnosis, APACHE II Score,{Knaus, 1985 #1112} Functional Comorbidity Index,{Groll, 2005 #1500} Charlson Comorbidity Index,{Charlson, 1994 #1501} past medical history of chronic hypertension or congestive heart failure). We inferred ideal body weight by calculating the weight required to have a BMI of 22 for given height.[12] Volume of intravenous fluids and information on potential vasopressor-associated adverse outcomes was collected daily. Specifically, we did not screen for adverse outcomes, but local research staff prospectively sought for occurrences of myocardial injury (as defined by troponin elevation), and limb necrosis, bowel ischemia, gastric intolerance, major hemorrhage, venous thromboembolism, and vasopressor extravasation (as defined by clinical team). Use of mechanical ventilation, renal replacement therapy and vasopressors was assessed daily and we inferred ventilator, renal replacement therapy and vasopressor-free days as days alive and free of the given therapy within the first 28 days.[13] Except for survival status, which we collected at the time of hospital discharge, we censored data collection at 28 days.

### Statistical analyses

MAP values during hours on vasopressors are described both hourly by patient and by patient average which averages all hours on vasopressors within each patient to obtain a single value per patient. These MAP values were approximately normally distributed and are described using means and standard deviations. We reported medians and quartiles for variables that were strongly skewed including all duration variables.

We depicted the MAP while on vasopressors over time by graphing the patient specific trajectories with a non-parametric LOESS smoother of the hourly median and quartiles overlaid.{Cleveland, 1993 #1502} We presented boxplots to depict the patient-averaged MAP by site. The association between total hourly vasopressors and patient average MAP was also presented graphically overall and by chronic hypertension status with a scatterplot distinguishing hospital survivors from decedents and regression lines superimposed.

To measure potential associations between baseline characteristics and MAP, we conducted single predictor and multivariable linear regression analyses with age, chronic hypertension, congestive heart failure and site as independent variables and the patient-averaged MAP while receiving vasopressors as the dependent variable. Within the linear regression model, the site effect was tested by a 5 degree of freedom F-test which equates to a one-way analysis of variance in the single predictor model.We selected these variables *a priori* on the basis of clinical plausibility. Fisher’s exact test was used to compare hospital mortality between patients with and without chronic hypertension.

The analysis was performed using SAS version 9.4 (SAS Institute Inc, Cary, NC, USA). The dataset is available as a Supporting Information file (S1 Study Dataset).

## Results

From March 18, 2014 to July 2, 2014, we enrolled 60 patients in 1 rural and 5 urban participating centres (median number of acute care beds 475; quartiles 440, 550). All centres were university-affiliated and enrolled patients from mixed medical-surgical intensive care units. For three patients, vasopressors were discontinued within hours before any data collection, and data were lost for one patient, leaving 56 patients in this analysis. The mean (SD) age was 64 (14) years and the APACHE II score was 25 (8). Half of the patients (29, 52%) had chronic hypertension and a minority (9, 16%) had congestive heart failure (Table 1).

**Table 1:** Baseline characteristics.

Baseline characteristics	n = 56
Age (years)	63.9±14.2 (27–88)
Sex	
Male	36 (64%)
Female	20 (36%)
Weight (kg)	81.5±25.6 (35–145)
Ideal body weight (kg)*	62.2±8.9 (22.9–80.3)
Body mass index	28.5±8.6 (11.2–44.2)
APACHE II Score	25.0±7.9 (11–43)
Primary Admission Diagnosis	
Sepsis (all)	42 (75%)
Not otherwise specified	19 (34%)
Respiratory	13 (23%)
Urinary	5 (9%)
Abdominal	4 (7%)
Soft tissues	1 (2%)
Cardiac Failure	5 (9%)
Chronic obstructive pulmonary disease	3 (5%)
Hypoxemic respiratory failure not otherwise specified	1 (2%)
Bowel resection	2 (4%)
Drug overdose	1 (2%)
Adrenal insufficiency	1 (2%)
Encephalitis	1 (2%)
Functional comorbidity index	1.4±1.1 (0–4)
Charlson comorbidity index	1.9±1.9 (0–9)
Days in hospital before enrollment	0.9 [0.7 to 3.6] (0.1–56.7)
Chronic Hypertension	29 (52%)
Congestive heart failure	9 (16%)

Reported as mean±SD (range), median [Q1, Q3] (range), or n(%).

* Weight required to have a BMI of 22 for given height.

The median (1^st^ quartile, 3^rd^ quartile) duration of vasopressor therapy was 43 (23, 84) hours and the median duration of mechanical ventilation was, 5 (3, 9) days. Thirty-two percent of patients received renal replacement therapy while in the ICU. ICU mortality was 39%, 28-day mortality was 41% and hospital mortality was 48%. At 28 days, we observed a median (quartiles) of 12 (0, 23) mechanical ventilator-free days, 22 (4, 28) renal replacement therapy-free days and 20 (1, 25) vasopressor-free days.

**Fig 1:**
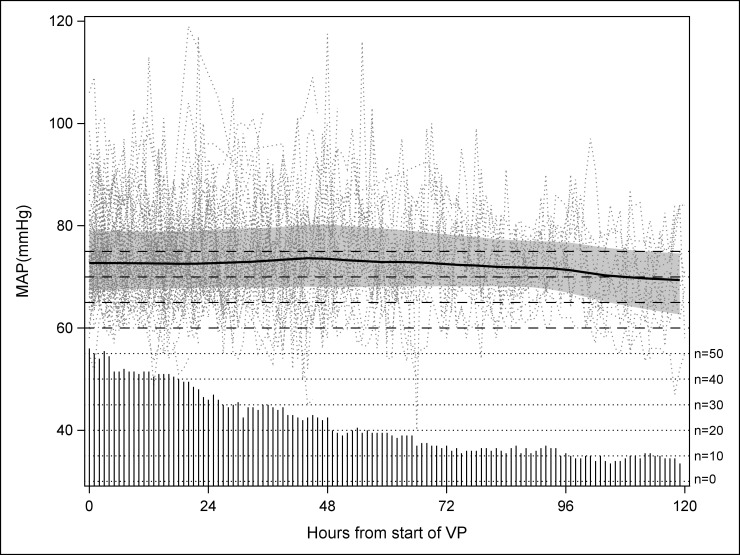
MAP over time for hours on vasopressors. Dots depict the individual patient hourly MAP measurements. The shaded area and thick line represent the LOESS smoothed hourly interquartile range and median respectively. The needles represent the number of patient contributing to each hour as labeled on the right axis.

Fig 1 displays hourly MAP values during vasopressor therapy. Five per cent of MAP values were not recorded. Of the 4384 hourly MAP measurements, the average (SD) MAP was 72 mmHg (10). The interquartile ranged from 66 to 78. The patient-averaged (SD) MAP while on vasopressors was 75 mmHg (6). A breakdown of the MAP by range is provided in Table 2. MAP was at least 65 mmHg on 81% of all patient-hours on vasopressors and the patient-averaged MAP was above 70 mmHg for 75% of the patients. There was no obvious time trend in MAP over the first 5 days of vasopressor use.

**Table 2:** MAP frequency by range.

MAP range (mmHg)	Patient-hours; n (%)(n = 4384)	Hours by patient*; median [Q1, Q2](n = 56)	Patient-averaged MAP**; n (%)(n = 56)
<60	265 (6%)	1 [0, 4]	0 (0%)
60 to <65	577 (13%)	3 [0, 11]	4 (7%)
65 to <70	921 (21%)	6 [2, 20]	10 (18%)
70 to <75	1016 (23%)	19 [3, 17]	20 (36%)
≥75	1605 (37%)	17 [5, 32]	22 (39%)

* These are the hours individual patients spent in the given MAP range.

** This is the is the n (%) of patients whose average MAP fell in the given range.

**Fig 2:**
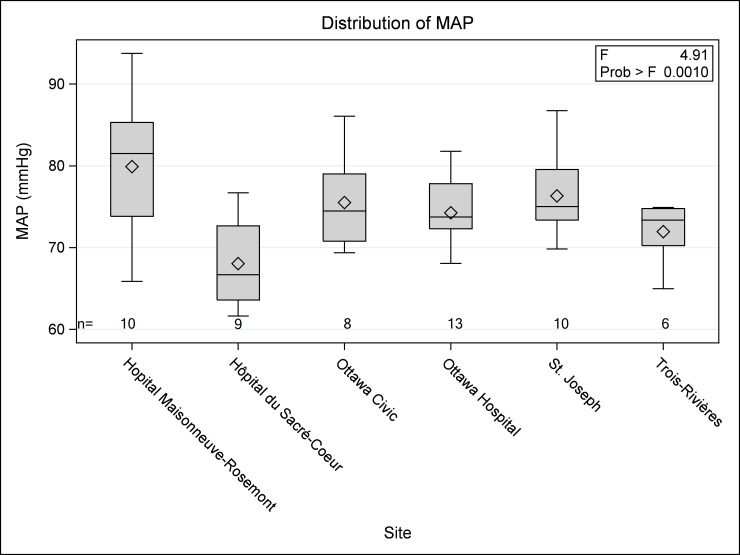
Distribution of patient averaged MAP for hours on vasopressors by site.

The patient averaged MAP while receiving vasopressors was not significantly associated with baseline characteristics (Table 3) but did vary by center (Fig 2).

**Table 3:** Association between baseline characteristics and mean arterial pressure target.

	Single predictor models		Adjusted combined model	
Predictor	Beta (95% CI)	p-value	Beta (95% CI)	p-value
Age (per decade)	-1.2 (-2.4, 0.0)	0.046	-0.7 (-1.8, 0.4)	0.19
Chronic hypertension	-1.8 (-5.2, 1.7)	0.31	-1.9 (-5.1, 1.2)	0.21
Congestive heart failure	2.1 (-2.6, 6.8)	0.38	-1.3 (-6.2, 3.6)	0.60
Site	See Fig 2	0.001	12.3 (6.6,17.9)*	0.002

Adjusted multiple predictor model R^2^ = 0.40 (p = 0.001)

*Expected mean difference between highest and lowest site.

We confirmed that residuals were approximately normal, co-linearity was trivial, and no single observation was influential enough to alter conclusions.

On days on which they received vasopressors, patients also exhibited a positive fluid balance while fluid output exceeded input after vasopressor discontinuation (Table 4).

**Table 4:** Fluid balance on days with and without vasopressors.

	Days with VP			Days without VP		
	(patients = 56)			(patients = 41)		
	Median	Q1	Q3	Median	Q1	Q3
EN/oral intake	518	208	1005	1046	696	1507
Crystalloids	2072	1395	2870	999	563	1382
Starches	0	0	0	0	0	0
Albumin 25%	0	0	16	0	0	29
Albumin 5%	5	0	254	0	0	0
Blood products	0	0	106	0	0	37
Total IV	2133	1395	3469	1124	594	1439
Total Input	2941	2228	4067	2046	1433	2835
Urine Output	1246	266	2613	1738	580	2697
NG Output	31	0	135	0	0	0
Other Output	0	0	159	2	0	180
Total Output	2102	1130	2920	2574	1570	3218
Fluid Balance	981	-219	2059	-494	-998	506
Patients Positive	40 (71.4%)			18 (43.9%)		
Patients Negative	16 (28.6%)			23 (56.1%)		

VP-Vasopressors, Q1-First quartile, Q3-Thrid quartile.

**Fig 3:**
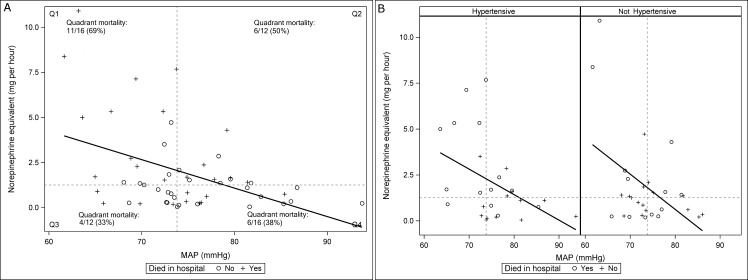
Association between average hourly MAP and vasopressor dose. a) All patients. b) Patients separated by chronic hypertension status. Vertical and horizontal dashed lines represent, respectively, the median-patient averaged arterial pressure and vasopressor dose.

For days during which vasopressors were received, myocardial injury occurred in 31 (55%) patients, cardiac arrhythmias in 9 (16%), limb necrosis in 1 (2%), bowel ischemia in 1 (2%), gastric intolerance in 6 (11%), major hemorrhage in 3 (5%), and venous thromboembolic events in 1 (2%). No patient suffered from vasopressor extravasation. Hospital mortality rates were not significantly different among patients with and without chronic hypertension (45% vs. 52%, respectively, p = 0.79). Fig 3 depicts the association between patient-averaged MAP (x axis) and vasopressor dose (y axis) overall and in patients with and without chronic hypertension.

## Discussion

In this multicenter, prospective observational study, the patient-level average MAP while receiving vasopressors for vasodilatory hypotension was 75 mmHg, which is approximately 10 mmHg above current recommendations[8] and stated practices.[9] Moreover, our results do not support the notion that clinicians tailor vasopressor therapy to individual patient characteristics such as underlying chronic hypertension. MAP achieved while receiving vasopressors varied by site, suggesting that vasopressor dosing may be influenced by local practice patterns rather than individual patient characteristics.

The high vasopressor-induced MAP values we observed in this study suggest that nurses and physicians take great care to avoid under-dosing vasopressors, perhaps related to concerns about the potential harmful effect of hypotension.[14] Our study highlights an opportunity to better monitor and control vasopressor therapy for severe hypotension. The potential risks associated with excessive vasopressor therapy may be under-appreciated or under-valued, relative to the potential risks of hypotension. This underscores the need to carefully evaluate the consequences of different target MAP values—and actual MAP values—when using vasopressor therapy.

Reducing unnecessary and undesired exposure to medications that have consistently been associated with adverse cardiac events both in observational studies [7] and in clinical trials [15] may improve patient outcomes. In addition, translating emerging data from clinical trials of vasopressor dosing strategies to clinical practice will require more accurate knowledge of actual MAP values achieved in practice, as well as tighter control over MAP targets. In contrast to stated practices, MAP targets below 70 mmHg are inconsistent with usual care and could be considered experimental. Similarly, our results challenge the notion that it is standard to treat hypertensive and non-hypertensive patients differently.

Limitations of our study include a small sample size. However, given the standard deviation of the patient-averaged MAP (6.4 mmHg), 56 patients allow us to estimate the mean patient-average MAP to within 1.7 mmHg with 95% certainty. For the secondary objective of determining if MAP was associated with chronic hypertension, congestive heart failure or age, this sample size provides 80% power at a two-sided alpha of 5% to detect a 5 mmHg difference between patients with or without chronic hypertension, and more generally 80% power to detect any predictor that explained at least 15% of the variance in the patient average MAP. Thus we believe our sample size is adequate to detect moderate to strong predictors of MAP. The fact that we did not collect information on the prescribed targets prevents us from determining if the MAP values were intentionally maintained above 65 mmHg by the treating physician or if there were discrepancies between prescribed targets and MAP achieved. We also did not ascertain, in patients who transitioned to palliative care but who remained on vasopressors, at which time the goals of care were reassessed. This would be expected to bias our analyses towards lower MAP values and therefore, our analyses may underestimate the actual MAP while receiving vasopressors. This study was conducted before the investigators of the SEPSISPAM trial reported lower rates of renal replacement therapy in chronically hypertensive patients treated with higher MAP targets (80 to 85 mmHg).[15] It is unclear whether observed practices would be different today since critical care physicians already stated that they aim for higher MAP values in chronically hypertensive patients before the publication of these results. Strengths of our study include the prospective identification of eligible patients, which increases the relevance of these findings to concurrent clinical trials of higher versus lower MAP targets, the multicenter design, and the detailed analysis of hourly vasopressor dose and MAP.

In summary, this study showed that the majority of the time during vasopressor therapy MAP was maintained between 65 to 80 mmHg with a mean patient-average of 75 mmHg at particpating centres. We found MAP varied significantly by site but was not significantly associated with age, underlying chronic hypertension or congestive heart failure.

## Supporting Information

S1 Study Dataset (XLS)
